# Valorization of Beach-Cast Biomass from the Invasive Seaweed *Rugulopteryx okamurae* on the Moroccan Coast: A Comparative Assessment of Extraction Conditions for Sodium Alginate Recovery

**DOI:** 10.3390/ijms27177898

**Published:** 2026-09-04

**Authors:** Khansae Kamal, Zahira Belattmania, Fouad Bentiss, Charafeddine Jama, Abdellatif Chaouti, Valérie Stiger-Pouvreau, Brahim Sabour

**Affiliations:** 1 Phycology, Blue Biodiversity and Biotechnology RU, Laboratory of Plant Biotechnology, Ecology and Ecosystem Valorization—CNRST Labeled Research Unit N°10, Faculty of Sciences, University Chouaib Doukkali, P.O. Box 20, El Jadida 24000, Morocco; kamal.khansae@ucd.ac.ma (K.K.); chaouti@ucd.ac.ma (A.C.); sabour.b@ucd.ac.ma (B.S.); 2 Laboratory of Catalysis and Corrosion of Materials, Faculty of Sciences, University Chouaib Doukkali, P.O. Box 20, El Jadida 24000, Morocco; fbentiss@gmail.com; 3 Materials and Transformations Unit, University of Lille, CNRS, INRAE, Centrale Lille, UMR 8207-UMET, F-59000 Lille, France; charafeddine.jama@univ-lille.fr; 4 University Brest, CNRS, IRD, Ifremer, LEMAR, IUEM, F-29280 Plouzane, France; stiger@univ-brest.fr

**Keywords:** sodium alginate, FT-IR, ^1^H NMR, invasive brown seaweed, *Rugulopteryx okamurae*, beach-casted biomass, Moroccan coastline

## Abstract

*Rugulopteryx okamurae* is an aggressively invasive seaweed whose proliferation along the Moroccan coastline represents both an ecological problem and an underexploited source of biomass. This work highlights the isolation of sodium alginate from *R. okamurae* under four combinations of acid pretreatment and alkaline (carbonate) extraction conditions. The extracted alginates were characterized by determining the extraction yield and analyzing their structural features using Fourier-transform infrared spectroscopy (FTIR) and proton nuclear magnetic resonance (^1^H NMR). Alginate yields ranged from 11 to ~16% (dry weight), and the highest yield 15.85 ± 0.66% dw was obtained with acidification treatments of 2 h at 40 °C and alkaline extraction at 60 °C over 5 h. FTIR analysis confirmed the sodium alginate structure and suggested the absence of detectable sulfate polysaccharide (fucoidan) contamination, as evidenced by the absence of a S=O band. ^1^H NMR analysis revealed a guluronate rich polymer within the four used protocols, highlighting its potential to form a strong and rigid gel. The present study highlights the alien *R. okamurae* as a viable source of high guluronate sodium alginate and clarifies how extraction conditions might shape its yields.

## 1. Introduction

**Figure 1:**
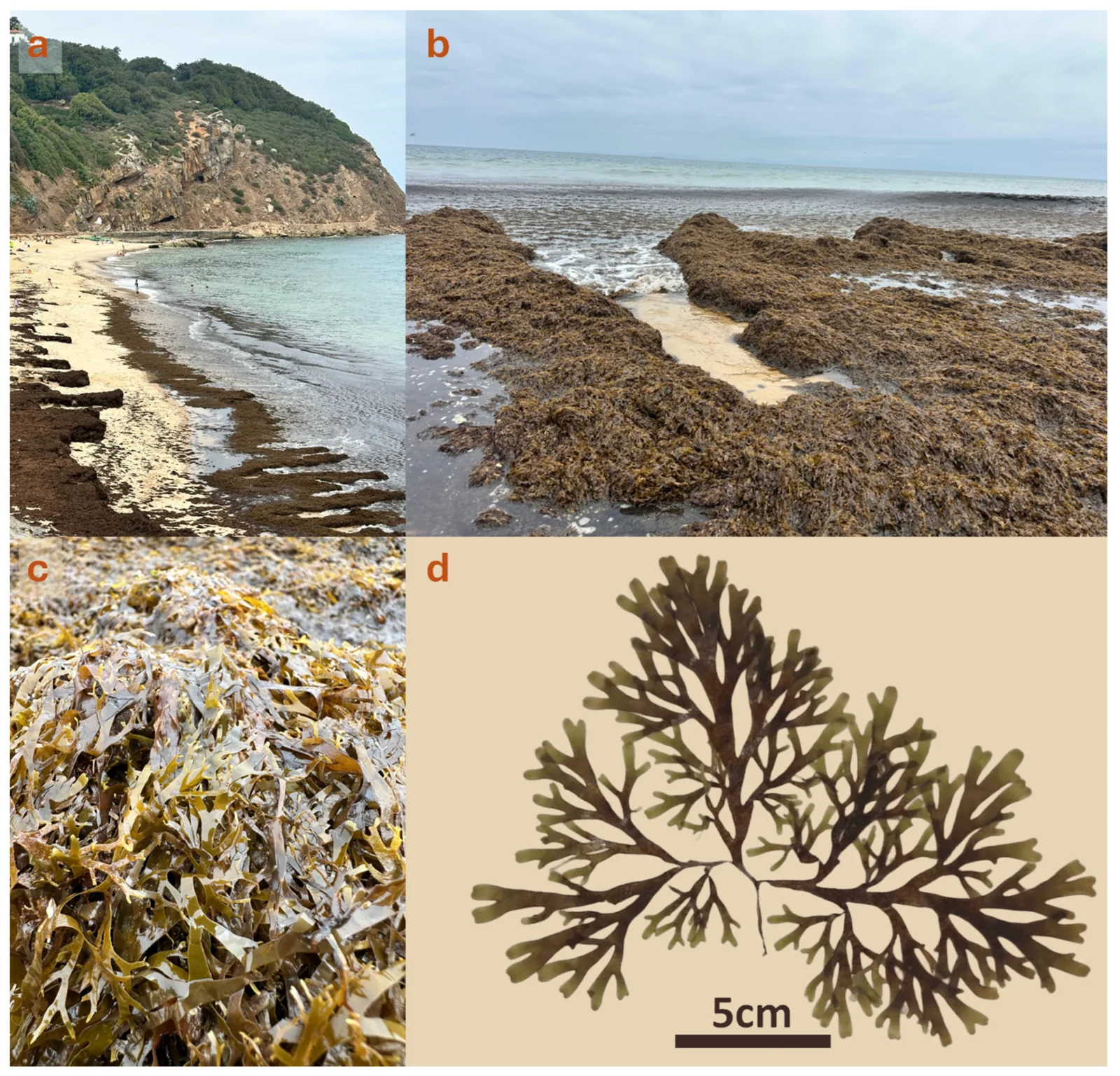
Sampling site and morphology of *R. okamurae*. (**a**,**b**) Field view of the intertidal zone at Merkala beach, Tangier (Morocco), showing accumulated biomass. (**c**) Field photograph of the collected specimen. (**d**) Representative specimen illustrating the branched morphology of *R. okamurae*.

Few marine invasions have unfolded as rapidly, or as visibly, as that of *Rugulopteryx okamurae* (E.Y. Dawson) (Dictyotales, Phaeophyceae), known natively in Japan as フクリンアミジ (Fukurin-amiji) [1,2]. Native to the northwestern Pacific, this brown macroalga was documented on the Moroccan Mediterranean and Atlantic coast [3,4]. *R. okamurae* has since become one of the most conspicuous invasive macroalgae in the region, forming dense benthic cover and generating substantial beach-cast accumulations along the shoreline (Figure 1). The rapid proliferation of invasive macroalgae can substantially alter native communities and ecosystem structure, with consequences for biodiversity and trophic interactions [5,6]. The severity of the invasion has prompted concern beyond the initially affected Spanish and Moroccan coasts: since 2022, the species has been recognized as a threat to marine ecosystems by the European Union and is now included in the European List of Invasive Alien Species (IAS) (EU 2022/2013) [5]. In just over a decade, *Rugulopteryx okamurae* has rapidly spread throughout the Strait of Gibraltar, the western Mediterranean, and adjacent Atlantic coastal areas. This significant proliferation has led to the establishment of dense populations that have negatively impacted autochthonous communities [6,7]. Furthermore, the continued expansion of *R. okamurae* also highlights the need for coordinated monitoring and regional management strategies to limit its spread and associated ecological impacts [5].

However, this remarkable abundance also makes the species particularly attractive as a potential biomass resource. Since biomass must be harvested as part of invasive species management efforts, its transformation into marketable products [8,9] could help offset the costs associated with controlling this invasive species. At the same time, the economic value of the harvested biomass could provide an additional incentive for its collection, thus reinforcing the ecological justification for its management [10].

The most promising target within this feedstock is alginate. It is the single most abundant component of *R. okamurae*, reported to be roughly one third of the dry biomass [11,12].

Alginate is a linear anionic polysaccharide built from (1 → 4)-linked β-D-mannuronic acid (M) and α-L-guluronic acid (G) residues, organized into homopolymeric M-blocks, homopolymeric G-blocks, and alternating MG-blocks [13]. The alginate sequence is a determining factor in the functional properties of this biopolymer. The proportion of the two residues, expressed by the M/G ratio, as well as their sequential arrangement along the polymer chain, determine whether the polymer forms a soft, flexible gel or a stiff, thermostable one [14]. In particular, G-blocks bind divalent cations through the “egg-box” mechanism. Consequently, guluronate-rich alginates generally form stronger and more rigid hydrogels, making them particularly suitable for encapsulation and structural applications [15]. Beyond that, it is a commercially established biopolymer used across the food, pharmaceutical, biomedical, and materials sectors [16]. Alginate is isolated from seaweed through an acid step, which converts the polymer into insoluble alginic acid and strips away acid-soluble impurities, followed by an alkaline (carbonate) treatment, which redissolves it as sodium alginate before precipitation. Each extraction step can be run gently or harshly, and the choice is not free. More severe alkaline conditions, such as higher temperatures and prolonged extraction times, can increase alginate recovery but can also promote chain breakage, leading to a reduction in molecular mass and potentially altering the material’s functional properties [14].

This study takes up both questions. Using a duplicated two-factor design that varies the acid and carbonate steps independently, we ask how extraction severity governs the yield and composition of *R. okamurae* alginate. In parallel, we characterize alginate from drifting biomasses on the Atlantic Moroccan coast as a source not previously described, combining FTIR, and ^1^H NMR. In doing so, we also confront a small but persistent puzzle: a reproducible NMR signal near 4.96 ppm that appears in *R. okamurae* alginate spectra across independent studies yet has never been identified, and which is a loose thread we set out to bound, if not yet fully resolve. Overall, the exploration of *R. okamurae* contributes to advancing scientific knowledge on the extraction and characterization of alginates. Furthermore, its valorization can offer potential environmental benefits by helping to reduce the ecological impacts associated with the accumulation and spread of this invasive species. In this context, the study of beach-cast *R. okamurae* in Morocco contributes both to the understanding of alginate extraction and to the valorization of an invasive species, thus illustrating how a problematic biomass can be turned into a useful resource with potential economic and environmental benefits.

## 2. Results and Discussion

### 2.1. Alginate Yield

The alginate yields obtained under the four protocols (PR1, PR2, PR3 and PR4) are summarized in Table 1. Yields ranged from 11.19 ± 0.52% (PR1) to 15.85 ± 0.66% (PR3), with the low standard deviations reflecting good reproducibility across independent extractions. Both extraction assumptions were satisfied prior to analysis (Shapiro–Wilk *p* = 0.259 and Levene’s *p* = 0.808), permitting parametric analysis.

Two-way ANOVA revealed that both steps significantly affected the yield, but to markedly different degrees. Carbonation had the dominant effect (F(1, 8) = 100.4, *p* < 0.001), while acidification contributed a smaller but significant effect (F(1, 8) = 18.8, *p* = 0.003); no significant interaction was found between the two factors (F(1, 8) = 1.33, *p* = 0.283). The absence of interaction indicates that the two extraction steps acted independently and additively. The highest yield was obtained when both acid and carbonate treatments were intensified (PR3), the lowest when both were mild (PR1), and the single factor protocols (PR2 and PR4) fell in between. This ranking by F-value identifies the carbonation step as the principal determinant of alginate recovery.

Pairwise comparisons (Tukey HSD) confirmed this pattern. PR3 yielded significantly more alginate than all other protocols (*p* < 0.05), whereas PR1 and PR4 (the two protocols sharing the mild carbonation step) did not differ significantly (*p* = 0.190). The fact that modifying the acidification step alone (PR1 versus PR4) failed to produce a significant increase, while intensifying the carbonation step markedly improved recovery, reinforces the predominant role of carbonation. This reflects the function of the carbonation step, in which sodium carbonate solubilizes alginate as sodium alginate; the heated, extended treatment likely promoted more complete conversion and release of the polymer from the algal matrix, increasing the extraction yield [17].

The predominance of the carbonation conditions agrees with previous studies investigating the optimization of the alginate extraction process. Using a response-surface approach, Rincón-Cervera et al. [12] identified elevated temperature and extended extraction time as optimal for alginate recovery from *R. okamurae*. Comparable optimal conditions (60 °C, 5 h) have been reported for *Sargassum* spp. [18]. The intensified carbonation applied here (60 °C, 5 h) corresponds to these conditions, and the present factorial analysis independently confirms, through direct comparison of discrete protocols, that it governs the increase in yield. Importantly, these differences in yield were achieved without altering the guluronate rich composition of the alginate (Section 2.2 and Section 2.3), indicating that the extraction can be intensified to maximize recovery from *R. okamurae* drift biomass while preserving the structural quality of the product. The alginate yields obtained in the present work fall within the range reported for the main alginophyte species on the Moroccan coast [16]. Similarly, the yields obtained for *R. okamurae* (11–16% dw) are consistent with the range of 3–32% dw previously reported for this species [19,20]. Furthermore, highest yield was achieved under the optimized conditions of PR3, in agreement with the extraction parameters reported by Rincón-Cervera et al. [12], from which this protocol was adapted.

### 2.2. FTIR Spectroscopy Analysis

**Figure 2:**
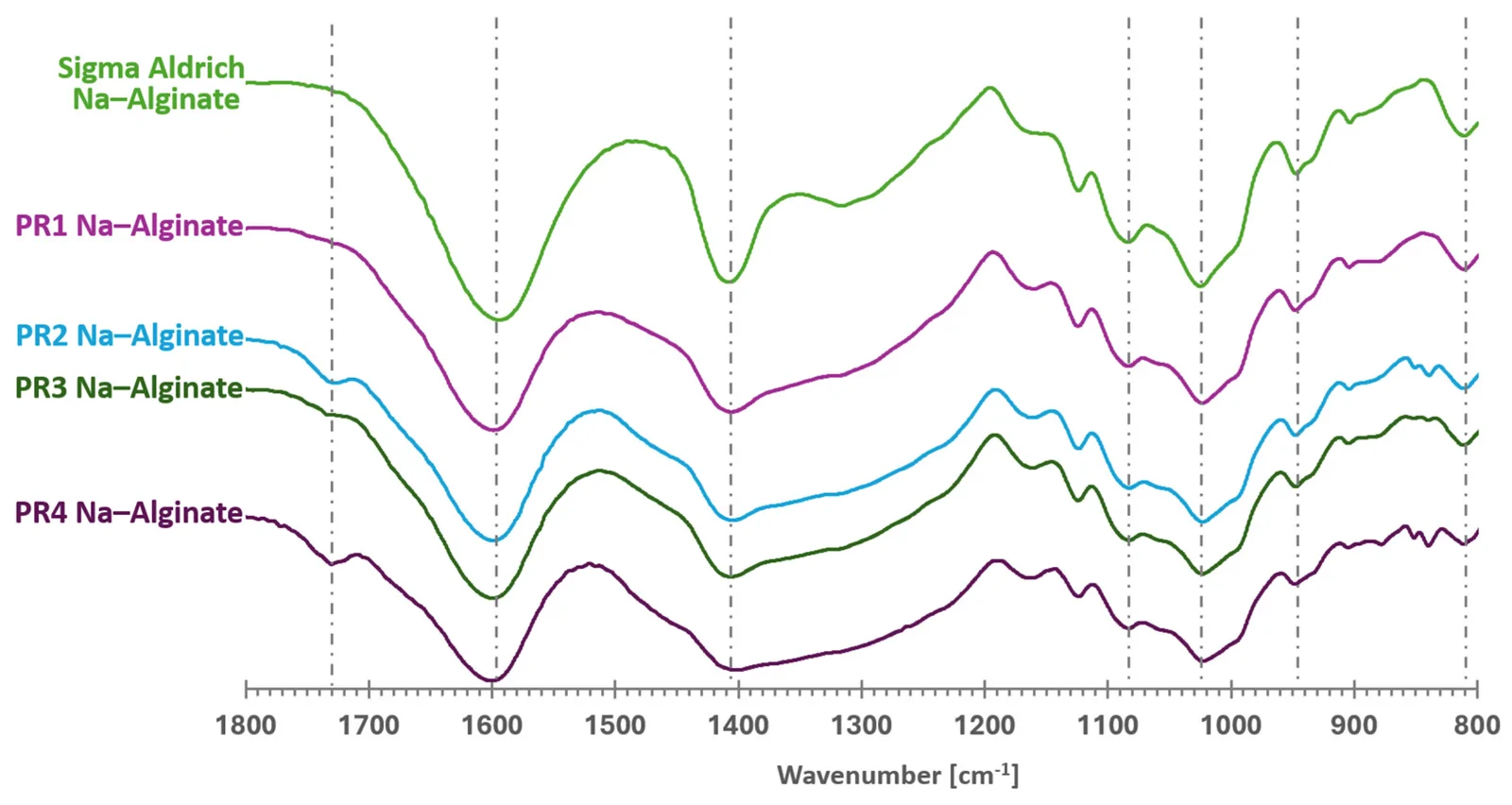
FTIR spectra of sodium alginate extracted under the four protocols (PR1–PR4) and of commercial sodium alginate (Sigma-Aldrich).

The functional groups of the extracted sodium alginates from beach-casted biomass of the invasive brown seaweed *R. okamurae* were characterized by FTIR, allowing comparison between all four PR1–PR4 protocols and assignment of the characteristic alginate absorption bands (Figure 2). Commercial Sigma-Aldrich sodium alginate served as a reference for band assignment. The absorption bands of sodium alginate are present in all five spectra, indicating that the target polysaccharide was obtained with each extraction protocol applied to *R. okamurae* biomass.

Asymmetric and symmetric stretching vibrations of the carboxylate (COO−) groups of uronic acid residues are assigned to the two most diagnostic bands, which are recorded at approximately 1594 cm^−1^ and 1405 cm^−1^ [21]. A systematic difference was observed in the carbonyl region between alginates obtained from the different protocols used. PR1 showed only the carboxylate bands, with no absorption in the 1710–1730 cm^−1^ region. This confirms that the alginate was recovered entirely in its deprotonated sodium salt form [16]. In contrast, PR2, PR3, and PR4 each exhibited an additional band at approximately 1728 cm^−1^, characteristic of the C=O stretching of protonated carboxylic acid (–COOH) groups. The presence of this band alongside the carboxylate bands stipulates that these alginate samples retained a fraction of non-neutralized carboxylic acid groups, reflecting incomplete total conversion to the sodium salt form. PR1 was the only protocol in which both the acidification and carbonation steps were performed at room temperature over 24 h, while each of PR2–PR4 involved a heated and shortened acidification and/or carbonation step. Thus, the appearance of the carboxylic acid ester (C=O) band at 1728 cm^−1^ appears associated with these modified conditions, which were less effective at achieving complete neutralization of the alginic acid. The C-O and C-C stretching of the pyranose ring are responsible for the bands at approximately 1080 and 1021 cm^−1^, respectively [21,22]. In the fingerprint region, all spectra exhibited a distinct band at 945 cm^−1^, corresponding to the C–O stretching vibration of uronic acid residues. Furthermore, the absorption band detected at 810 cm^−1^ has consistently been attributed to mannuronic acid residues [16,23].

Importantly, the alginates obtained using the four extraction protocols exhibited the same characteristic bands as the commercial standard. This indicated that the chemical identity and the principal functional groups of the alginate samples were not altered. The spectral profile was reproducible across all four protocols, indicating consistent alginate recovery under various extraction conditions. This reproducibility is advantageous for scale-up. This consistency is in agreement with published assignments for *R. okamurae* [12]. Furthermore, sulfated polysaccharide contamination was excluded by the absence of the 1230–1280 cm^−1^ band [24]. Meanwhile, FTIR is a simple, rapid, and low-cost technique that reliably confirms the functional groups of the extracted alginate, but it does not resolve the functional properties of the polymer. Therefore, ^1^H NMR spectroscopy is the recognized reference employed to determine the M/G ratio and the block composition of the extracted alginate [25].

### 2.3. ^1^H NMR Spectroscopy Analysis

**Figure 3:**
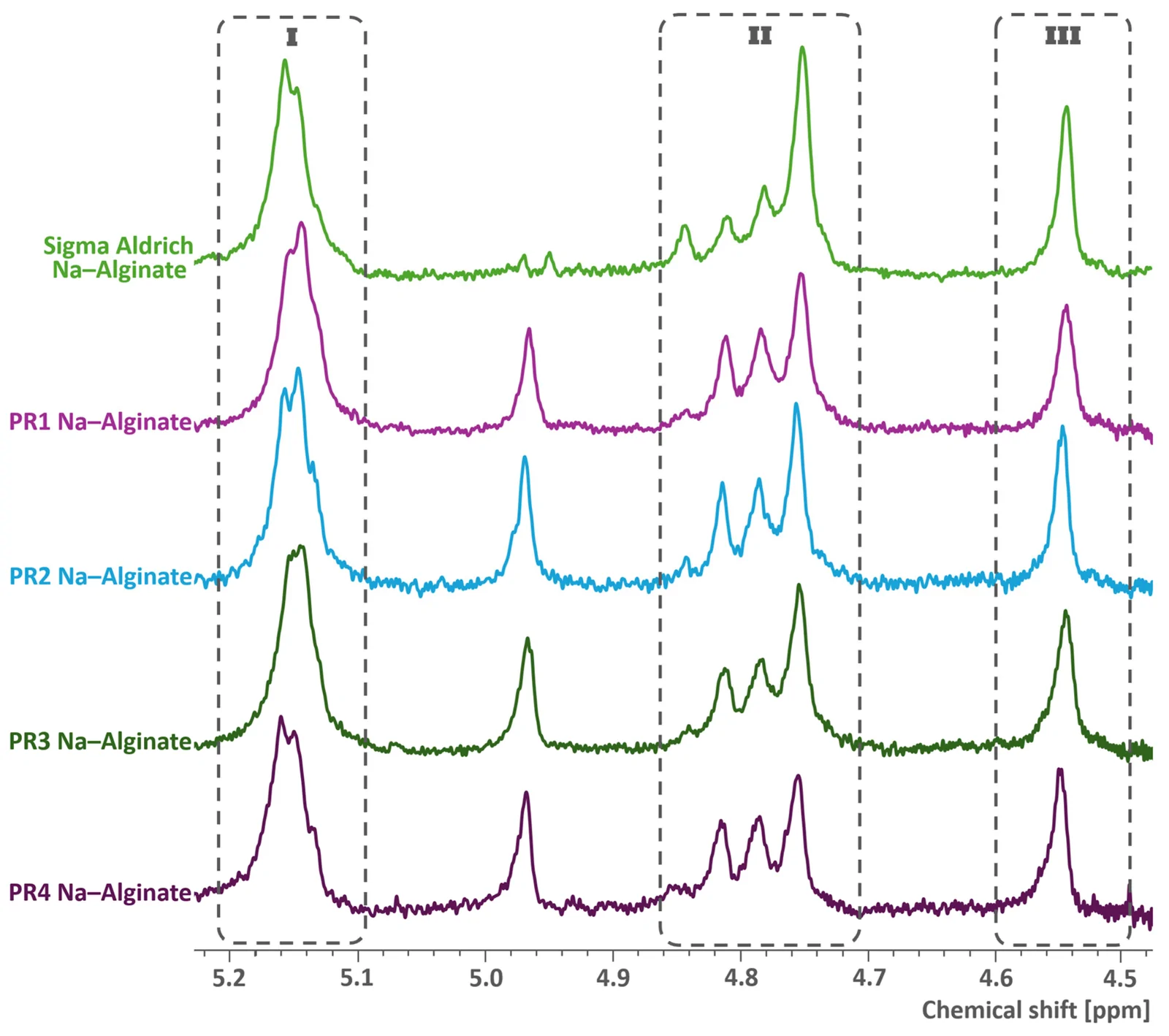
^1^H NMR spectra of commercial sodium alginate (Sigma-Aldrich) and alginates from the four protocols (PR1–PR4). I: signal attributed to anomeric hydrogen of guluronic acid (G). II: signals of H-1 on mannuronic acid (M) and H-5 on GM alternating blocks. III: signal corresponding to the H-5 of homopolymeric G residues.

The structural composition of the extracted alginate was determined by ^1^H-NMR spectroscopy recorded in D_2_O at 343 K. All four extracted alginates (PR1–PR4) and the commercial standard exhibited the three diagnostic anomeric signals of alginate (Figure 3): the guluronate anomeric proton (G-1) at ~5.1–5.2 ppm (I), the overlapping mannuronate anomeric proton (M-1) and H-5 of alternating GM blocks at ~4.74–4.85 ppm (II), and the H-5 of homopolymeric guluronate blocks at ~4.5–4.6 ppm (III) [25]. The spectral concordance between the extracted samples and the commercial standard confirmed the identity of the recovered polysaccharide as alginate.

The molar fractions of guluronate (FG) and mannuronate (FM) and the M/G ratio were derived from signals I–III, according to Grasdalen et al. [26] (Table 2). All four protocols yielded a markedly guluronate-rich alginate (FG = 0.75–0.82; M/G = 0.21–0.33), in every case exceeding the guluronate content of the commercial Sigma-Aldrich standard. The alginate remained G-rich across the four protocols despite substantial differences in acidification and carbonation conditions, although PR4 showed a lower M/G ratio (0.21) than the other protocols. The lower M/G ratio observed for PR4 may suggest an influence of the intensified acidification treatment; however, the present data are insufficient to distinguish a genuine process-related effect from experimental variation. No definitive mechanistic explanation is, therefore, proposed. Nevertheless, the consistently low M/G values across all protocols indicate the G-rich character is a prominent feature of the recovered alginate from this species. Acidification induces protonation of the carboxylate groups of alginate, yielding the comparatively insoluble alginic acid form. Subsequent treatment with Na_2_CO_3_ neutralizes these carboxylic acid groups, facilitating conversion to the water-soluble sodium alginate salt [14]. Variations in the acidification and carbonation steps, therefore, influence the extent of neutralization and the final salt form in which the polymer is recovered. This finding complements the FTIR results, where the modified protocols altered the degree of neutralization and, consequently, the relative protonated and sodium salt forms of the recovered alginate, without substantially affecting the polysaccharide backbone. Together, the two spectroscopic analyses show that the extraction conditions govern recovery and the final salt form as they allow extraction to be optimized for yield or purity without compromising its structural quality.

This guluronate rich profile places *R. okamurae* alginate within the G-rich end of the broad compositional range reported for brown algae. Alginate composition varies widely among species, and its molecular characteristics, particularly the M/G ratio and the distribution of M and G residues, strongly influence its physicochemical and functional properties, including viscosity and gel-forming behavior [13]. *Macrocystis pyrifera*, a major commercial source, is mannuronate-rich (≈62% M, 38% G) and forms soft, elastic gels, whereas *Laminaria hyperborea* is strongly guluronate-rich (≈45% M, 55% G) and forms rigid gels, with reported M/G values across sources spanning roughly from 0.4 to 1.63 [27]. The low M/G ratio obtained here places *R. okamurae* alginate toward the *L. hyperborea* end of this spectrum. This is the compositional class prized for strong-gelling and structuring applications [28]. Functionally, this is because the high guluronate content responsible for the formation of rigid and thermally stable Ca^2+^ mediated “egg-box” gels in *L. hyperborea* alginate is the same feature identified for *R. okamurae* in the present study. Meanwhile, *M. pyrifera* and *Ascophyllum nodosum* alginates, which contain higher portion of mannuronate, generally form softer and more elastic gels [27]. That alginate, which is from an invasive, otherwise drifting biomass, falls into the same G-rich category because the premium commercial source is a central outcome of this work and directly supports the valorization of *R. okamurae* as a raw material for alginate. This result supports the guluronate-rich character previously reported for the species, with low M/G ratios (0.47–0.88), strengthening the interpretation that the G-rich character is a reproducible feature of *R. okamurae* rather than a result of a single study distortion [11,12]. Some inter-study variation is, nonetheless, expected, as the M/G ratio varies with season, growing environment, geographic origin, and extraction method [14]. In addition to the overall M/G ratio, the sequence and distribution of M and G residues are important structural parameters that can influence alginate functionality, particularly its rheological and Ca^2+^ induced gelation behavior [13]. Diad-level sequence parameters and block lengths were not reported in the present study because the additional resonance at ~4.96 ppm falls outside the conventional three-region integration scheme and, therefore, precludes their reliable determination. The monomeric composition (FG, FM and M/G), however, remains unaffected, being derived from the well-resolved anomeric signals.

A reproducible resonance between the signals I and II at ~4.96 ppm was observed in the four obtained ^1^H-NMR spectra; this was also visible in previously reported *R. okamurae* alginate spectra but not assigned, or treated as an impurity [10,12]. This signal does not correspond to any of the three standard alginate diagnostic peaks. A plausible initial hypothesis was that the observed signal originated from a co-extracted polymer and could be originating from a co-extracted sulfated polysaccharide (e.g., fucoidan or a galactofucan) [29]. However, this hypothesis is not supported by the present FTIR data. Unlike commercial fucoidan, which typically exhibits a very distinct sulfate ester ν(S=O) absorption in the 1230–1260 cm^−1^ region, it was not detectable and was not observed in any of the extracted alginate samples. This finding argues against the presence of significant amounts of highly sulfated fucoidan. These data do not support the presence of highly sulfated fucoidan as the origin of this resonance. A residual desulfated fucan (sulfates are, potentially, stripped during alkaline extraction, which would erase the FTIR signature while leaving the sugar visible via NMR) cannot be excluded based on the present evidence alone, but is considered unlikely to account for an intense signal. Its reproducibility across different protocols, collection sites, and alginates of differing M/G argues against a random impurity or a variable reducing end signal and points, instead, to a consistent component or structural feature of *R. okamurae* alginate. Accordingly, definitive assignment of this resonance cannot be established from the present one dimensional ^1^H NMR data. Such an assignment would require two-dimensional NMR (2D NMR), such as ^1^H-^13^C HSQC and/or ^1^H-^1^H COSY experiments, to establish the relevant proton–carbon and proton–proton correlations and determine whether the observed resonance can be assigned to the alginate backbone.

### 2.4. Toward Valorization and IAS Management

The valorization of *R. okamurae,* especially drifting biomass, offers a route to convert an environmental liability into an economic resource. Introduced to the Strait of Gibraltar most probably by ballast water and hull fouling, the specie has since expanded rapidly, displacing native benthic communities and generating substantial accumulations of stranded biomass along the coastline [30]. These strandings impose direct costs on coastal municipalities through collection and disposal, while the invasion itself has been associated with declines in native biodiversity, disruption of local artisanal fisheries, and adverse effects on coastal tourism [5]. Current management relies largely on the removal and landfilling or incineration of stranded biomass, an approach that is costly and generates no return.

Establishing a commercially viable alginate extraction process from this drift biomass reframes that management burden as a feedstock supply. The present results show that beach-casted *R. okamurae* yields guluronate rich alginate of a quality comparable to premium commercial sources, and that this quality is recovered consistently across a range of extraction conditions. A regional alginate industry based on this invasive material could, therefore, align two objectives that are usually treated separately: supporting invasive species management for local authorities, and supplying a value-added marine biopolymer within a circular, blue economy framework.

## 3. Materials and Methods

### 3.1. Sampling Site and Biomass Pretreatment

Drift biomass of *R. okamurae* (Figure 1) was collected on June 2025 along a large transect at the Tangier shoreline (35°47′40.75″ N 5°50′05.17″ W), Morocco, from recently drifting material retained in the shallow coastal water. The collected biomass was pooled and homogenized, followed by manual separation to remove visible epiphytes. Given the fragile nature of the biomass, the algal material was gently rinsed with tap water, followed by rinsing with deionized water, to eliminate surface-adhered debris. The initial moisture content of the freshly collected biomass was 80 ± 0.55% (*n* = 3). The biomass was then shade dried until a constant weight was reached.

### 3.2. Alginate Extraction and Experimental Design

Sodium alginate was extracted from *R. okamurae* following a procedure adapted from Calumong et al. [31], with the acidification and carbonation conditions based on the optimization reported by Rincón-Cervera et al. [12].

**Figure 4:**
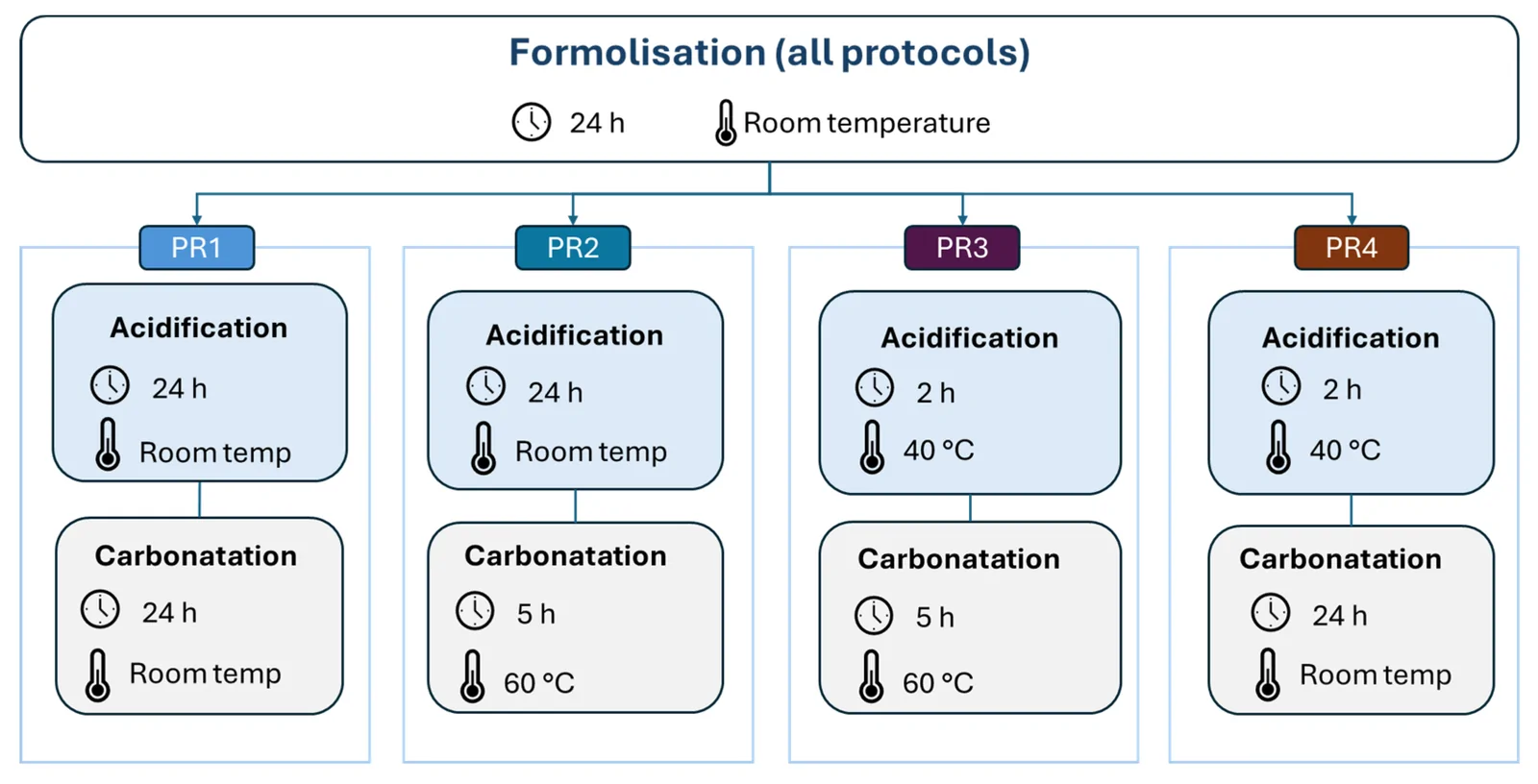
Flow chart of the four alginate extraction protocols (PR1–PR4) from the *R. okamurae* biomass.

In all protocols, 12.5 g of dried biomass was first subjected to formolisation using 2% formaldehyde at room temperature for 24 h, followed by washing with water. The biomass was subsequently acidified using 0.2 M HCl and subjected to carbonation using 2% Na_2_CO_3_. The reagent concentrations, biomass mass, and solution volume were kept constant across all protocols, while acidification and carbonation time and temperature were varied according to the experimental design. Four extraction protocols were tested using a 2 × 2 factorial design combining two acidification treatments (24 h at room temperature or 2 h at 40 °C) with two carbonation treatments (24 h at room temperature or 5 h at 60 °C). This yielded four protocols (PR1–PR4), as illustrated in Figure 4, where PR1 corresponds to the baseline Calumpong conditions (room-temperature acidification and carbonation), and PR3 represents the optimized conditions of Rincón-Cervera (heated, shortened acidification and carbonation), while PR2 and PR4 constitute intermediate conditions in which a single factor was modified, allowing the individual contributions of acidification and carbonation to be distinguished. Following carbonation, the sodium alginate was precipitated using ethanol 95%, washed by acetone 90%, and dried in an oven at 50 °C until a constant weight was achieved. Each extraction protocol was performed in triplicate. Extraction yield was expressed as a percentage of dry algal biomass (% dw).

### 3.3. FTIR Spectroscopy Analysis

FTIR spectra of the dried sodium alginate samples were placed on a Thermo Scientific Nicolet Impact 400D FT-IR spectrometer (Nicolet Instrument Co., Madison, WI, USA) in attenuated total reflectance (ATR) mode. Spectra were recorded over the 4000–600 cm^−1^ range at a resolution of 4 cm^−1^, with 32 scans averaged per sample. The resulting spectra were processed using OMNIC 7.1 software (Nicolet, Madison, WI, USA).

### 3.4. ^1^H NMR Spectroscopy Analysis

^1^H-NMR spectra of the sodium alginate samples, dissolved in D_2_O, were recorded at 343 K on a Bruker AV II 400 MHz spectrometer (9.4 T; proton Larmor frequency 400.33 MHz; Bruker Corporation, Billerica, MA, USA) equipped with a 5-mm triple-resonance broadband inverse (TBI) probe (Bruker Corporation, Billerica, MA, USA). Free induction decays were acquired with 16 K data points and a sweep width of 4800 Hz, averaging 32 transients. Presaturation was applied during the relaxation delay and mixing time to suppress the residual water signal. Prior to Fourier transformation, the data were apodized with a line-broadening factor of 0.5 Hz. A commercial sodium alginate (Sigma-Aldrich, Gillingham, UK) was used as the standard.

### 3.5. Statistical Analysis

Each extraction protocol used for this study was performed in triplicate (*n* = 3), and results are expressed as mean ± standard deviation. The effects of the acidification and carbonation conditions on extraction yield were evaluated using a two-way analysis of variance (ANOVA), with acidification and carbonation as fixed factors and their interaction included in the model. Prior to analysis, normality of the model residuals was verified using the Shapiro–Wilk test and the homogeneity of variances using Levene’s test. When significant effects were detected, pairwise comparisons between protocols were performed using Tukey’s honestly significant difference (HSD) test. Differences were considered statistically significant at *p* < 0.05. All analyses were carried out in R (version 4.6.1). The alginate M/G composition was determined from a single representative sample per protocol and is, therefore, reported descriptively, without statistical comparison.

## 4. Conclusions

This study characterized sodium alginate extracted from the brown invasive seaweed *R. okamurae*, using drift biomass collected from the Moroccan coast, and evaluated the influence of the extraction conditions on the recovered polymer. Four protocols, differing in their acidification and carbonation steps, were compared by extraction yield, FTIR, and ^1^H-NMR. The FTIR analysis confirmed the successful extraction of alginate using all four protocols, with spectra closely matching that of the commercial standard. A systematic difference was observed in the carbonyl region, indicating that the extraction conditions influenced the degree of neutralization. No sulfate absorption was detected, suggesting the absence of significant co-extracted sulfated polysaccharides. ^1^H-NMR analysis showed that the extracted alginate is guluronate rich (M/G ≈ 0.3). Notably, this composition remained consistent across all used protocols despite the substantial differences in extraction conditions, demonstrating that the guluronate-rich character is an inherent property of the biomass rather than a consequence of a particular extraction method. This compositional stability is advantageous for processing as it allows the extraction parameters to be adjusted according to yield and processing requirements without compromising the structural characteristics of the recovered alginate. The consistently low M/G ratio indicates a G-rich alginate composition, similar to G-rich commercial alginate sources. However, important physicochemical and functional properties, including molecular weight distribution and rheological behavior should be investigated in future works. These findings support the valorization of *R. okamurae* drift biomass. Although this invasive species currently represents an ecological and economic burden along invaded coastlines, it could serve as a viable feedstock for alginate, aligning invasive-species management with a circular, blue-economy approach. Further work should focus on the complete sequential characterization of the polymer, including diad frequencies and block-length distribution, which could not be reliably resolved here, as well as on the molecular weight and gelling performance of the extracted alginate, to fully establish its suitability for specific industrial applications.

## Figures and Tables

**Table 1:** Yield of extracted alginate from *R. okamurae* under the four extraction protocols (PR1–PR4).

Parameter	PR1	PR2	PR3	PR4
Yield (% dw)	11.19 ± 0.52 ^c^	14.07 ± 0.33 ^b^	15.85 ± 0.66 ^a^	12.22 ± 0.67 ^c^

Values are expressed as mean ± standard deviation (*n* = 3). Different superscript letters within a row indicate statistically significant differences among extraction protocols (Tukey HSD, *p* < 0.05).

**Table 2:** ^1^H-NMR composition of alginate extracted from *R. okamurae* under the four protocols (PR1–PR4) compared to the commercial sodium alginate.

Parameter	PR1	PR2	PR3	PR4	Commercial Standard (Sigma-Aldrich)
FG	0.75	0.77	0.75	0.82	0.68
FM	0.25	0.23	0.25	0.18	0.32
M/G	0.33	0.30	0.33	0.21	0.46

## Data Availability

The raw data supporting the conclusions of this article will be made available by the authors on request.
